# Research Status and Trends of Gut Microbiota and Intestinal Diseases Based on Bibliometrics

**DOI:** 10.3390/microorganisms13030673

**Published:** 2025-03-17

**Authors:** Xiao Sun, Jiancheng Zhai

**Affiliations:** 1 Natural Reserve Planning and Research Institute, East China University of Technology, Nanchang 330013, China; 2 School of Earth Sciences, East China University of Technology, Nanchang 330013, China; 3 College of Animal Science and Technology, Jiangxi Agricultural University, Nanchang 330029, China

**Keywords:** gut microbiota, intestinal diseases, bibliometric, hotspots, trends

## Abstract

Gut microbiota plays an important role in gut health, and its dysbiosis is closely related to the pathogenesis of various intestinal diseases. The field of gut microbiota and intestinal diseases has not yet been systematically quantified through bibliometric methods. This study conducted bibliometric analysis to delineate the evolution of research on gut microbiota and intestinal diseases. Data were sourced from the Web of Science Core Collection database from 2009 to 2023 and were scientometrically analyzed using CiteSpace. We have found that the number of annual publications has been steadily increasing and showing an upward trend. China and the Chinese Academy of Sciences are the country and institution with the most contributions, respectively. *Frontiers in Microbiology* and *Nutrients* are the journals with the most publications, while *Plos One* and *Nature* are the journals with the most citations. The field has shifted from focusing on traditional descriptive analysis of gut microbiota composition to exploring the causal relationship between gut microbiota and intestinal diseases. The research hotspots and trends mainly include the correlation between specific intestinal diseases and gut microbiota diversity, the mechanism of gut microbiota involvement in intestinal diseases, the exploration of important gut microbiota related to intestinal diseases, and the relationship between gut microbiota and human gut health. This study provides a comprehensive knowledge map of gut microbiota and intestinal diseases, highlights key research areas, and outlines potential future directions.

## 1. Introduction

Trillions of symbiotic microorganisms colonize the mammalian gut and interact with the host in physiological processes, such as cell proliferation and immune response, even co-evolving with the host over thousands of years [1,2]. The diversity, stability, and resilience of gut microbiota, as well as their symbiotic interactions with hosts, have been formed during long-term co-evolution, which determines the complex interaction between gut microbiota and gut health [3,4]. Gut microbiota plays various functions in the host, including intestinal development, homeostasis, and protection against pathogenic bacteria. Gut microbiota can provide a wide range of metabolic functions for the host, which comes from anaerobic fermentation of exogenous undigested dietary components such as short-chain fatty acids (SCFAs), or endogenous substances produced by microorganisms and the host such as bile acid (BA) metabolites [5,6,7]. Gut microbiota is a key regulatory factor in intestinal digestion. Gut microbiota also prevents the invasion and colonization of pathogenic bacteria by maintaining the integrity of the intestinal epithelium, as well as competing processes such as nutrient metabolism, pH regulation, and antimicrobial peptide secretion [8]. In addition, symbiotic gut microbiota and their products can play a crucial role in regulating the development, homeostasis, and function of innate and adaptive immune cells [9].

Growing investigations have revealed that gut microbiota is a key mediator in maintaining the health of the host’s gut, playing a complex and indispensable role in the host’s physiological and pathological state [10]. Dysbiosis of gut microbiota is associated with the pathogenesis of various intestinal diseases (referring to diseases that affect the structure of the small and large intestines), such as irritable bowel syndrome (IBS), inflammatory bowel diseases (IBD), celiac disease (CD), colorectal cancer (CRC), and antibiotic-associated diarrhea (AAD) [11]. IBS is one of the most common gastrointestinal diseases, and the role of gut microbiota has been extensively studied [12]. IBD aggregates idiopathic, chronic, and relapsing inflammatory conditions of the gastrointestinal tract including ulcerative colitis (UC), Crohn’s disease (CRD), and indeterminate colitis (IC), which can be distinguished by the location of gastrointestinal inflammation [13,14]. Abundant evidence suggests that gut microbiota may be involved in the pathogenesis of IBD, such as the interaction between the melanocortin system and gut microbiota [15,16,17]. CD is a chronic intestinal inflammatory disease caused by an abnormal immune response to the dietary gluten protein in genetically susceptible individuals. Variations in the gut microbiota may play an important role in the pathogenesis of CD [18,19]. As the third most common cause of cancer mortality in the world, it has been confirmed that there are significant differences in gut microbiota between CRC patients and healthy volunteers [20,21]. The variations in the gut microbiota, such as a decrease in butyrate producers and an increase in opportunistic pathogens, constitute the main imbalance in the gut microbiota structure of CRC patients [21]. AAD is also one of the common intestinal diseases, which is a common adverse reaction of antibiotic treatment caused by the destruction of gut microbiota. One of its reasons is that Clostridioides difficile (formerly Clostridium) has reduced resistance to antibiotics, leading to colorectal infections [22]. Overall, the relationship between gut microbiota and various intestinal diseases is inseparable and complex, and their interactions are still widely, deeply, and precisely studied.

At present, the research on gut microbiota and intestinal diseases has attracted more and more attention from researchers around the world, and specific research has been carried out, resulting in many research achievements [23,24,25,26]. However, there is a lack of comprehensive and systematic analysis. Therefore, we use bibliometric methods to gain a more intuitive, comprehensive, and systematic understanding of the development history, research hotspots, and future trends in the field of gut microbiota and intestinal diseases. Bibliometrics uses mathematical and statistical methods to quantitatively analyze the distribution, structure, quantity, and evolution of publication information, which is of great value in understanding the status, trends, researchers, institutions, countries, and determining future research hotspots and academic frontiers in the research field. Bibliometrics has been widely applied in the fields of gut microbiota and its association with Alzheimer’s disease, lung diseases, epigenetics, and many others, providing decision support for the advancement of scientific research [27,28,29,30]. As a commonly used analytical tool in bibliometrics, CiteSpace software is a citation visualization literature analysis tool based on scientific metrology and data visualization theory, which can effectively display the status and trends of a certain discipline or knowledge field in a specific period [31,32]. We used CiteSpace software to conduct a systematic and scientific analysis of publications related to the field of gut microbiota and intestinal diseases, focusing on the number of publications, countries, institutions, authors, journals, keywords, co-cited authors, co-cited journals, and co-cited references. This study aims to comprehensively and systematically understand the relationship between gut microbiota and intestinal diseases, reveal research hotspots and development trends, and provide a solid foundation and reliable reference for scholars in this field.

## 2. Materials and Methods

### 2.1. Data Source and Retrieval Strategy

The data source was obtained from the Web of Science Core Collection (WoSCC), which is a multidisciplinary comprehensive database with a complete citation network and one of the most representative commonly used databases in bibliometric analysis. Considering its reliability and wide-ranging coverage, we selected the WoSCC database as the data source of the research object. The following retrieval strategy was used to gather all possibly pertinent publications based on title and abstract: TI = (gut OR intestine *) AND TI = (microb * OR flora OR bacteria *) AND AB = (intestinal disease). To avoid deviation errors, two researchers independently retrieved and identified the data sources on 19 June 2024 and ultimately reached a consensus. Since papers published in 2024 during the retrieval cannot represent the status of the entire year, papers published in 2024 were excluded.

**Figure 1:**
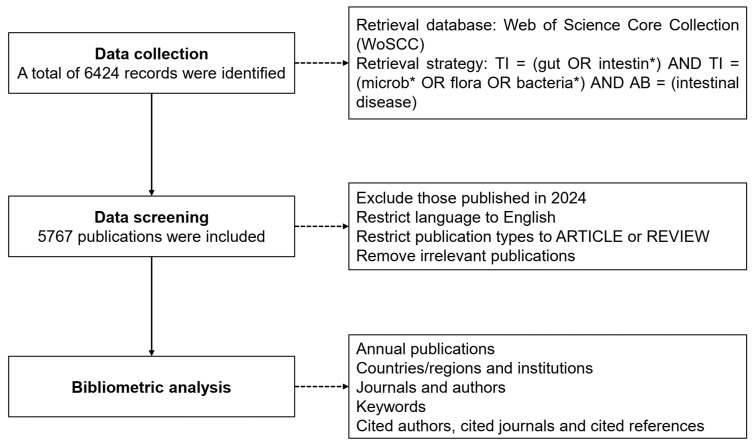
Flowchart of literature filtering and data analysis.

We set the language to English to minimize potential comprehension biases caused by language differences. Meanwhile, we specified the type of publications to include only ARTICLE and REVIEW, while excluding other types of articles such as editorials, conference proceedings, and letters. This was performed to ensure the consistency and reliability of our data analysis. In addition, we further filtered the literature based on specific keywords and topics related to gut microbiota and intestinal diseases to ensure the relevance of the included articles. Finally, a total of 5767 publications were obtained and exported in plain text format for subsequent analysis (Figure 1). The original data includes comprehensive information such as title, abstract, authors, institutions, journals, country/region, keywords, etc.

### 2.2. Data Analysis

Based on research requirements and software features, we used Microsoft Office Excel (Version 2010, Redmond, Washington, DC, USA), CiteSpace (Version 6.3.R1, Drexel University, Philadelphia, PA, USA), and Scimago Graphica (Version 1.0.35, SRG S.L. Company, Granada, Spain) software for data management and analysis. Microsoft Office Excel software can manage data, compile annual publications and journals, and create relevant tables and pictures. CiteSpace software is a citation visualization literature analysis tool based on scientific metrology and data visualization theory, which can automatically analyze and generate citation networks, calculate citation frequency, author collaboration, keyword distribution, and display this information in visualization charts [33,34]. To ensure the accuracy and completeness of data, CiteSpace was used for visualization and bibliometric analysis after removing duplicate entries. Following the guidelines of prior studies [35,36,37], we used CiteSpace software to analyze and create collaborative, co-occurrence and co-citation networks. The collaborative analysis function was used to analyze the collaborative networks of countries/regions, institutions, and authors. The co-occurrence analysis function was used to analyze the co-occurrence network of keywords, the clustering function was used for keyword clustering and displayed using the timeline view function, and the burst term function was used to analyze keyword bursts. The co-citation analysis function was used to analyze the co-citation relationships of cited authors, cited journals, and cited references. Based on the analysis data of countries/regions using CiteSpace software, Scimago Graphica software was used to create an international cooperation network diagram to more directly display the geographical distribution of publications.

## 3. Results

### 3.1. Analysis of Annual Publications and Trends in Publications

**Figure 2:**
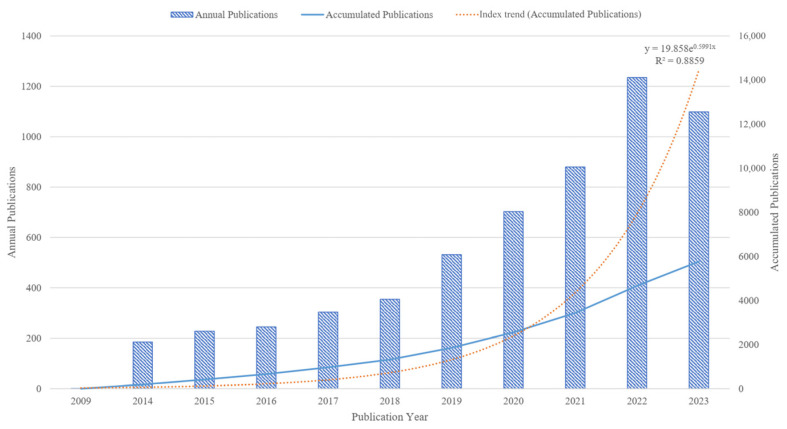
Distribution and trends of publications in the gut microbiota and intestinal diseases field.

The annual and accumulated publications reflect the general situation and development tendency of the gut microbiota and intestinal diseases field. A total of 5767 publications have been published since 2009 (Figure 2). Overall, the number of publications in the gut microbiota and intestinal diseases field is increasing and rapidly developing, from 1 publication in 2009 to 1099 publications in 2023, indicating that this field is receiving increasing attention from researchers. Since 2018, the number of publications has rapidly increased at a rate of around 200 per year, reaching a peak of 1235 publications in 2022. In addition, linear regression analysis was conducted on the publication time and cumulative publication, with R^2^ = 0.8859. The fitting effect of the model is good, which conforms to the scientific index growth law proposed by Price, that is, various scientific indicators increase exponentially over time [38]. This means that the annual publications in this field have a continuous upward trend.

### 3.2. Analysis of Countries/Regions and Institutions

**Figure 3:**
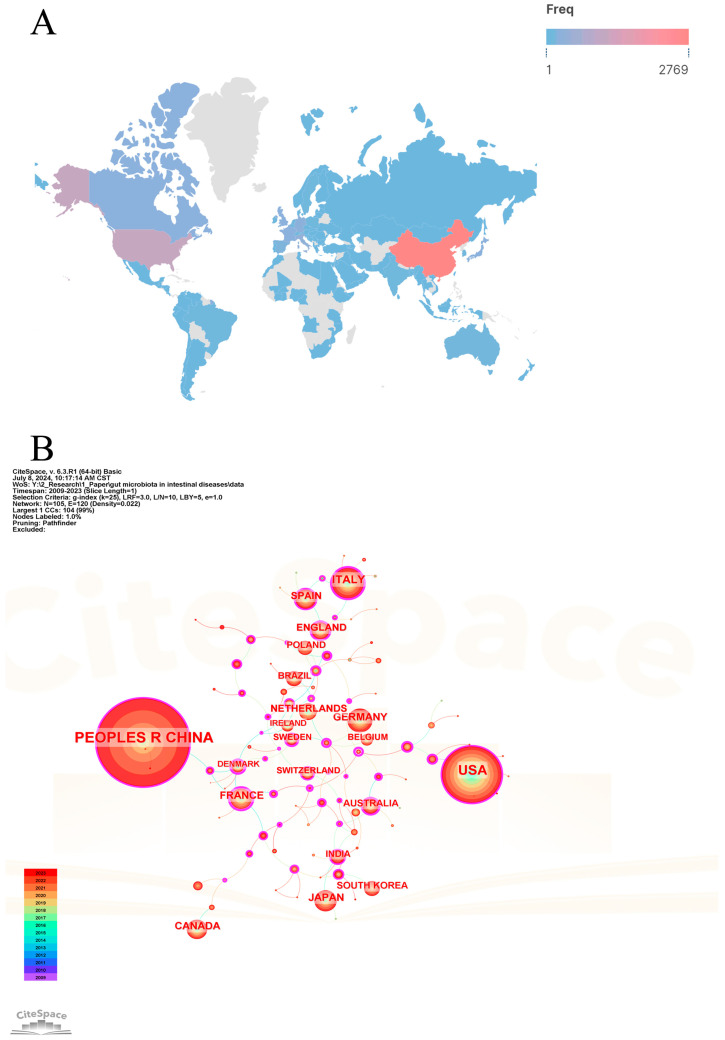
The visualization map of countries/regions analysis. (**A**) Geographic distributions of countries/regions. (**B**) Cooperative relationships of countries/regions.

Since 2009, 101 countries/regions have participated in the research of gut microbiota and intestinal diseases. Table 1 lists the top 20 countries/regions in terms of the number of publications. Figure 3A shows the global geographical distribution of publications, with different colors representing the frequency of publications in different countries/regions. Figure 3B shows the cooperation networks of countries/regions. China ranks first with a publication frequency of 2818, far higher than the second-ranked USA (1187), followed by Italy (350), Germany (241), and Japan (208) with lower frequencies. Cooperation among countries/regions is positively correlated with centrality. Among the top 20 countries/regions, France (0.5), Denmark (0.43), Australia (0.33), Sweden (0.27), and the United Kingdom (0.23) have the highest centrality. China and USA have the most publications in this field, but their centralities are not high, implying that their cooperation with other countries is not close and there is less international cooperation. This may be related to most research being conducted in the form of domestic cooperation, but it could also be attributed to their leadership in leading the development of the field. On the contrary, although the number of publications in other countries/regions is not very prominent, it demonstrates strong international cooperation relationships.

**Figure 4:**
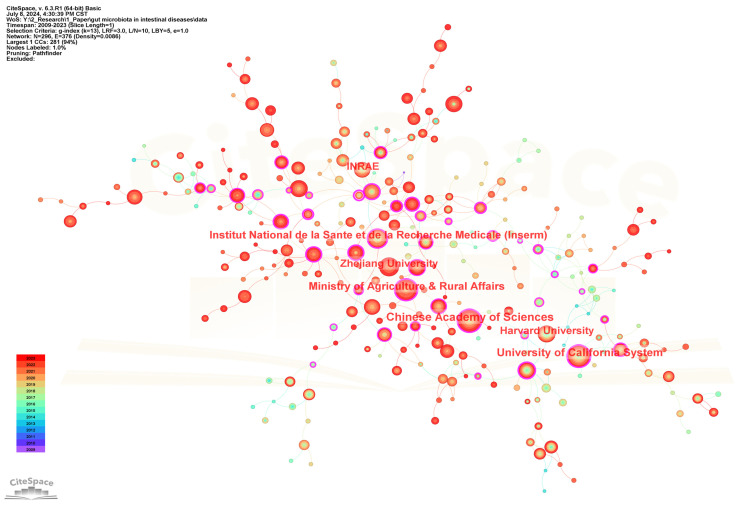
The visualization network map of institutions collaboration.

296 institutions contributed to the field of gut microbiota and intestinal diseases research. Table 2 lists the top 20 institutions in terms of the number of publications. Figure 4 shows the cooperation networks of institutions. The five institutions with the highest publication frequency are Chinese Academy of Sciences (154), University of California System (147), Institut National de la Sante et de la Recherche Medicale (INSERM) (111), Ministry of Agriculture and Rural Affairs (110), and Zhejiang University (102). In addition, Ministry of Agriculture and Rural Affairs (0.33), Université Paris Cité (0.31), Chinese Academy of Agricultural Sciences (0.3), Institut National de la Sante et de la Recherche Medicale (INSERM) (0.25), and Chinese Academy of Sciences (0.24) are the five institutions with the highest centrality, representing the strongest cooperation intensity. Meanwhile, the top 20 institutions belong to China, France, Italy, and USA. Among them, 12 institutions belong to China, with the Chinese Academy of Sciences having the highest publication frequency (154). Four institutions belong to France, with the highest frequency of publications being from Institut National de la Sante et de la Recherche Medicale (INSERM) (111). Three institutions belong to USA, with University of California System having the highest publication frequency (147). One institution belonging to Italy is Catholic University of the Sacred Heart (59).

### 3.3. Analysis of Journals and Authors

Articles related to the research of gut microbiota and intestinal diseases were published in 1194 journals. Table 3 lists the top 20 journals in terms of the number of publications and displays the journal impact factor (IF), category quartile, top journal, journal citation reports (JCR) category, and country. The top 20 journals published a total of 1831 publications, accounting for 31.75%. They all belong to Q1 and Q2 of the Journal Citation Reports (JCR), with 16 in Q1. Top journals usually refer to journals that are widely recognized as having high impact and high academic standards in a specific discipline, with 9 out of the top 20 journals belonging to the TOP journals. From the perspective of IF, the average IF of the top 20 journals is 6.4, with *Gastroenterology* (25.7) having the highest, followed by *Microbiome* (13.8) and *Gut Microbes* (12.2). The top 20 journals belong to six countries, including 8 in Switzerland, 5 in the USA, 4 in the United Kingdom, and 1 each in China, France, and the Netherlands. The journal with the highest number of publications is *Frontiers in Microbiology* (Q2, IF = 4.0, Top journal), with 198 publications, followed closely by *Nutrients* (180 publications, Q1, IF = 4.8), *Frontiers in Immunology* (179 publications, Q1, IF = 5.7), and *Frontiers in Cellular and Infection Microbiology* (132 publications, Q1, IF = 4.6) ranks fourth.

**Figure 5:**
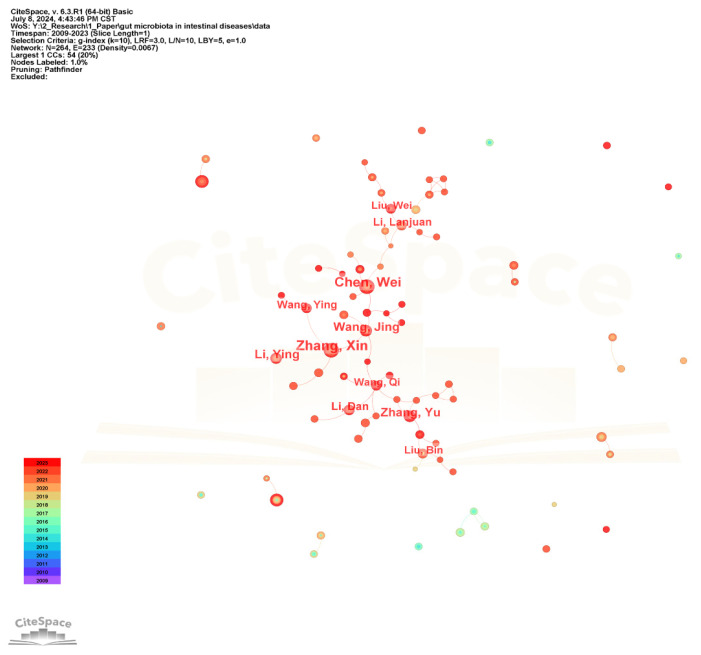
The visualization network map of authors’ collaboration.

The collaborative network of authors in the gut microbiota and intestinal diseases field is shown in Figure 5. The author nodes are relatively small and the frequency is low, with the highest being only 28. The top 10 authors with larger nodes are all Chinese, such as Zhang Xin, Chen Wei, Wang Jing, and Zhang Yu, forming a large author group centered around them and having close research cooperation relationships.

### 3.4. Keywords Analysis

**Figure 6:**
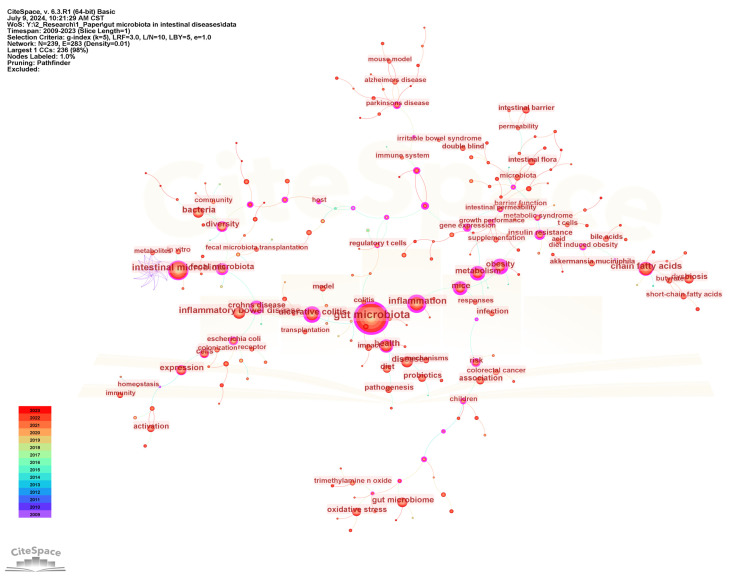
The visualization map of keyword co-occurrence network.

**Figure 7:**
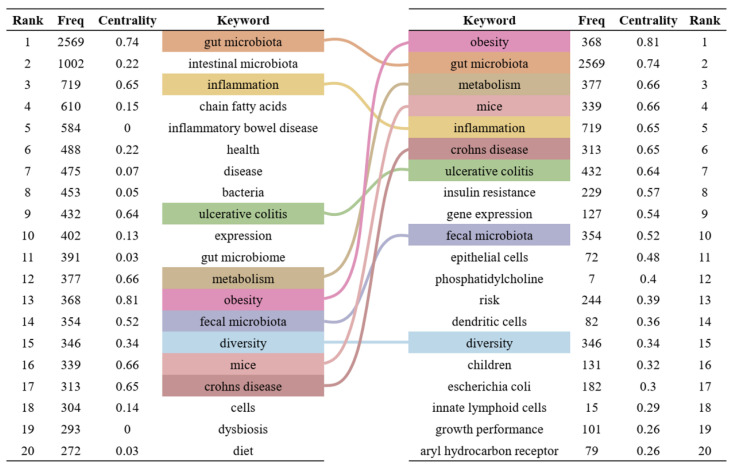
Top 20 keywords with the highest frequency and centrality. On the left are the top 20 keywords sorted by frequency, and on the right are the top 20 keywords sorted by centrality. The middle lines represent the common keywords on both sides.

Keywords are highly refined and summarized research content in publications. We constructed a keyword co-occurrence network (Figure 6) and analyzed the top 20 keywords with the highest frequency and centrality (Figure 7) to intuitively reveal the research history and trends in the field of gut microbiota and intestinal diseases. In the keyword co-occurrence network, the larger the node, the higher the frequency of the keyword. The top 10 most frequent keywords are gut microbiota (2569), followed by intestinal microbiota (1002), inflammation (719), chain fatty acids (610), inflammatory bowel disease (584), health (488), disease (475), bacteria (453), ulcerative colitis (432), and expression (402). The keywords with the highest centrality, representing the strongest cooperation density, are obesity (0.81), gut microbiota (0.74), metabolism (0.66), mice (0.66), inflammation (0.65), Crohn’s disease (0.65), ulcerative colitis (0.64), insulin resistance (0.57), gene expression (0.54), fecal microbiota (0.52). In addition, there are nine keywords that are both the top 20 with the highest frequency and the top 20 with the highest centrality, namely gut microbiota, inflammation, ulcerative colitis, metabolism, obesity, fecal microbiota, diversity, mice, Crohn’s disease (Figure 7).

The information on gut microbiota associated with specific intestinal diseases was listed in Table 4. The gut microbiota associated with IBS were mainly Firmicutes, Bacteroidetes, and Actinobacteriota, such as *Bacteroides dorei*, *Ruminococcus* gnavus, etc. The gut microbiota associated with IBD were mainly Firmicutes, Bacteroidetes, and Proteobacteria, such as *Faecalibacterium prausnitzii*, *Escherichia* coli, etc. The gut microbiota associated with CD were mainly Firmicutes and Bacteroidetes, such as *Dialister invisus*. The gut microbiota associated with CRC were mainly Fusobacteria, Proteobacteria, and Bacteroidetes, such as *Fusobacterium nucleatum*, *Escherichia coli*, *Bacteroides fragilis*, *Morganella* morganii, etc. The gut microbiota associated with AAD were mainly Firmicutes, Verrucomicrobia, Bacteroidetes, such as genera *Enterococcus*, *Eubacterium*, *Akkermansia*, *Alistipes*, and *Actinomyces*.

**Figure 8:**
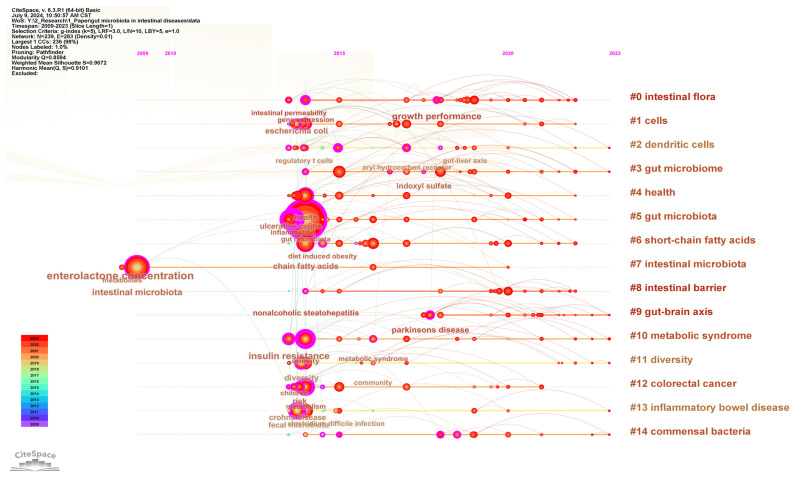
The timeline visualization map of keywords clustering analysis.

To understand the research forefront in the field of gut microbiota and intestinal diseases, keyword clustering analysis was performed and presented in the timeline visualization map (Figure 8). The keywords are clustered into 15 clusters, namely #0 intestinal flora, #1 cells, #2 dendritic cells, #3 gut microbiome, #4 health, #5 gut microbiota, #6 short-chain fatty acids, #7 intestinal microbiota, #8 intestinal barrier, #9 gut–brain axis, #10 metabolic syndrome, #11 diversity, #12 colorectal cancer, #13 inflammatory bowel disease, and #14 commensal bacteria. Except for clusters #7 intestinal microbiota starting from 2009 and #9 gut–brain axis starting from 2018, all other clusters started from 2014. Clusters #0 intestinal flora, #3 gut microbiome, #5 gut microbiota, and #7 intestinal microbiota are different uses of gut microbiota. Clusters #12 colorectal cancer and #13 inflammatory bowel disease are the most extensively studied intestinal diseases in this field. Clusters #1 cells, #2 dendritic cells, #4 health, #6 short-chain fatty acids, #8 intestinal barrier, #9 gut–brain axis, #10 metabolic syndrome, #11 diversity, and #14 commensal bacteria are the research focuses on the interaction between gut microbiota and host gut diseases.

**Figure 9:**
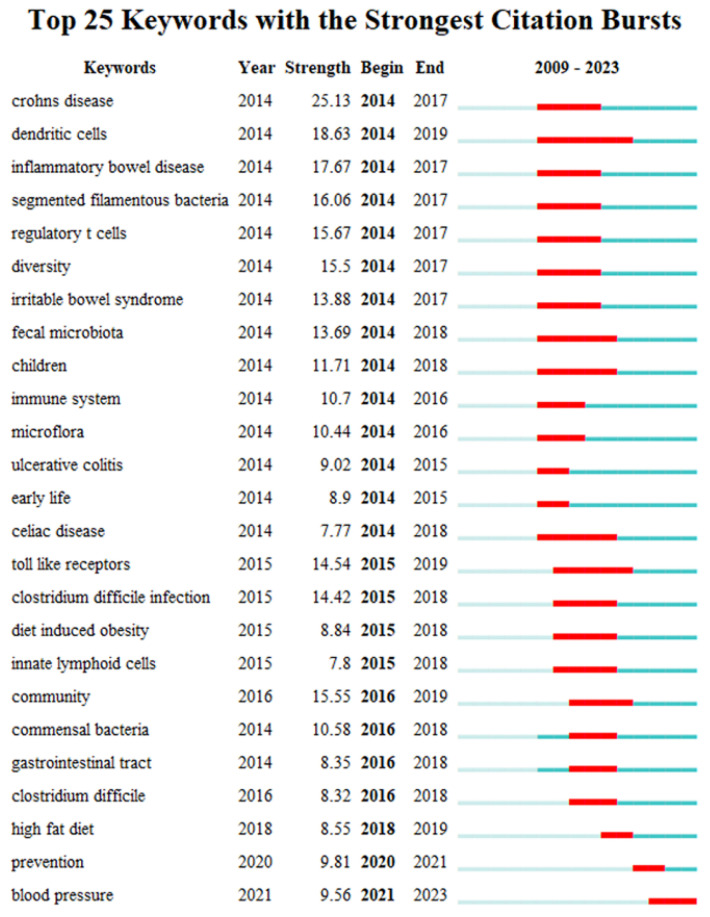
The top 25 keywords with the strongest citation bursts.

Keyword bursts can reveal a sudden increase in research content over a period, which may indicate future research trends [54]. Figure 9 shows the top 25 keywords with the strongest citation bursts in the field of gut microbiota and intestinal diseases. The red line in the figure represents the duration of the keyword exploded. From 2009 to 2023, the keywords have gradually shifted from “crohns disease”, “dendritic cells”, “inflammatory bowel disease”, “segmented filamentous bacteria”, “regulatory t cells”, “diversity”, “irritable bowel syndrome”, “ulcerative colitis” to “community”, “commensal bacteria”, “gastrointestinal tract”, “clostridium difficile”, and then to the current “high fat diet”, “prevention” and “blood pressure”. This indicates that the interaction between gut microbiota and intestinal diseases, the exploration of important gut microbiota related to intestinal diseases, and the relationship between gut microbiota and human intestinal health are current and future research priorities.

### 3.5. Co-Citation Analysis of Cited Authors, Cited Journals, and Cited References

**Figure 10:**
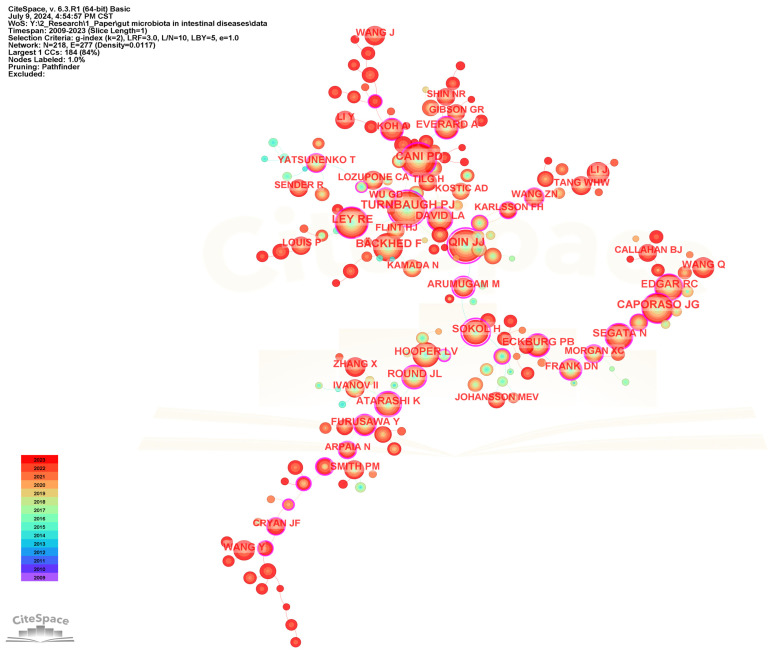
The visualization network map of cited authors.

To identify the most influential authors in the field of gut microbiota and intestinal diseases, a collaborative network of cited authors has been performed (Figure 10), and the top 20 most frequently cited authors are presented in Table 5. Turnbaugh PJ is the most frequently cited author with 887, followed by Cani PD (799), Qin JJ (771), Ley RE (700), and Backhed F (654). Qin JJ (0.92) and Sokol H (0.88) have the highest centrality among the top 20 most frequently cited authors, representing their influence and importance in the field.

**Figure 11:**
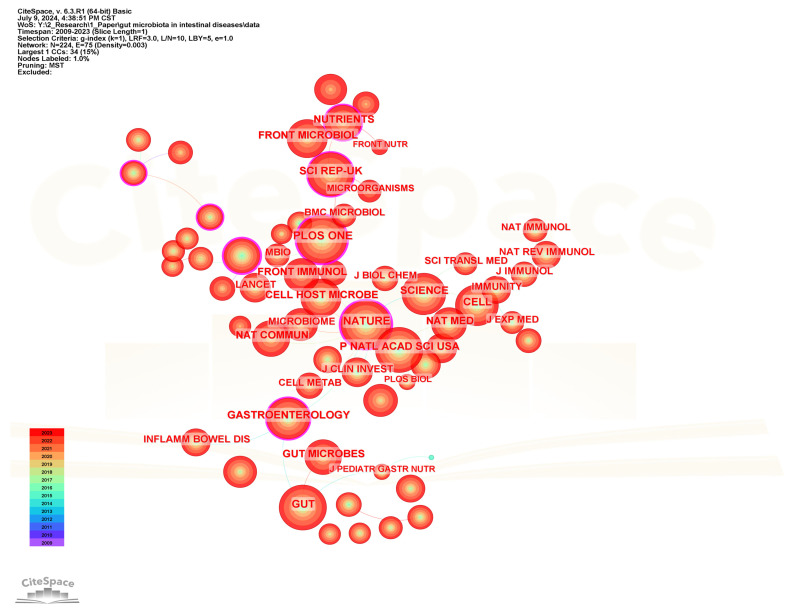
The visualization network map of cited journals.

Co-citation analysis of cited journals can help identify the journals with the highest citation frequency in the field of gut microbiota and intestinal diseases. Figure 11 shows the visualization network diagram of the cited journals, and Table 6 lists the top 20 journals with the highest citation frequency. *Plos One* is the journal with the highest citation frequency and the highest centrality among the top 20 frequently cited journals, although its impact factor is the lowest (IF = 2.9), indicating that it has a high influence in this field. Following closely in terms of citation frequency are *Nature*, *Gut*, *Proceedings of The National Academy of Sciences of The United States of America*, and *Science*. Among the top 20 frequently cited journals, *Lancet* has the highest impact factor (IF = 98.4) and ranks 20th in citation frequency. It is internationally recognized as one of the top four comprehensive medical journals. Meanwhile, the top 20 cited journals also include three recognized top-tier journals, namely *Nature*, *Science*, and *Cell*. In addition, there are also other journals with an impact factor greater than 20, such as *Nature Medicine* (58.7), *Nature Reviews Gastroenterology & Hepatology* (45.9), *Gastroenterology* (25.7), *Gut* (23.0), and *Cell Host & Microbe* (20.6).

**Figure 12:**
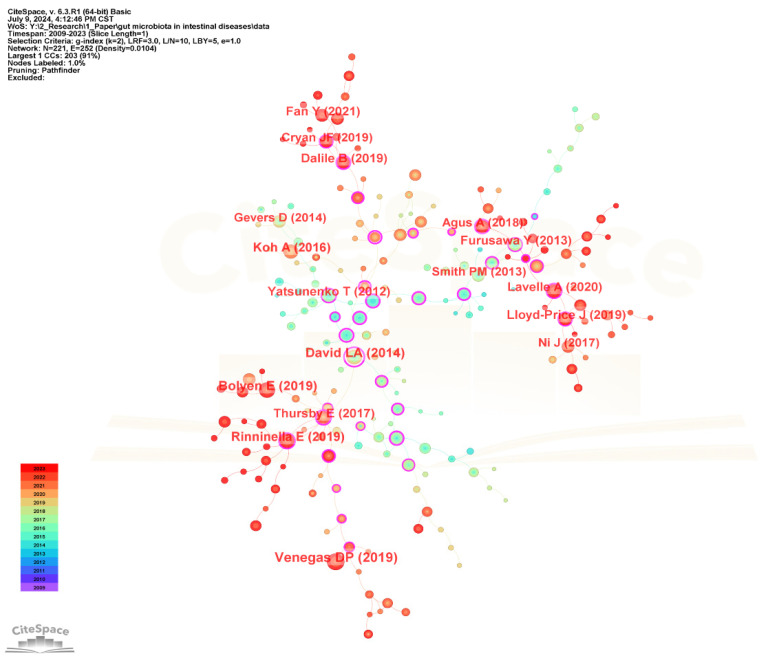
The visualization network map of cited references.

The visualization network diagram of the cited references is shown in Figure 12, and the top 20 most frequently cited references are listed in Table 7. The most frequently cited reference is “Short Chain Fatty Acids (SCFAs)—Mediated Gut Epithelial and Immune Regulation and Its Relevance for Inflammatory Bowel Diseases” published by Venegas DP in *Frontiers in Immunology* in 2019. It presents an overview of microbial short-chain fatty acids production and their effects on the intestinal mucosa with specific emphasis on their relevance for inflammatory bowel diseases (IBD) and discusses the therapeutic potential of SCFAs for IBD [55]. The second-ranked cited reference is “Reproducible, interactive, scalable and extensible microbiome data science using QIIME 2” published by Bolyen E in *Nature Biotechnology* in 2019, which developed a new microbiome analysis platform QIIME 2 to meet the needs of big data and reproducible analysis [56]. The third-ranked cited reference is “Diet rapidly and reproducibly alters the human gut microbiome” published by David LA in *Nature* in 2014 [57].

Among the top 20 most frequently cited references, six cited references studied the interaction between gut microbiota and inflammatory bowel diseases (Ulcerative colitis and Crohn’s disease), namely the 1st, 6th, 7th, 14th, 15th, and 19th cited references. There are seven cited references studied on how to affect host health by regulating gut microbiota metabolites (short-chain fatty acids and tryptophan), namely the 1st, 5th, 6th, 10th, 12th, 13th, and 17th cited references. There are six cited references studied on gut microbiota in human health or diseases, namely the 3rd, 4th, 8th, 9th, 11th, 18th, and 20th cited references. There are two cited references studied on the role of gut microbiota in the gut–brain axis, namely the 12th and 13th cited references. One cited reference is about the application of QIIME 2 tool in microbiome data, which is the 2nd cited reference.

## 4. Discussion

In this study, we retrieved 5767 publications related to gut microbiota and intestinal diseases through the Web of Science Core Collection database. Various bibliometric tools, including CiteSpace and Scimago Graphica, were employed to comprehensively analyze critical indicators such as countries/regions, institutions, journals, authors, keywords, cited authors, cited journals, and cited references in the research field. The number of publications has been steadily increasing every year and shows a general upward trend. Especially after 2018, it has grown at a rate of about 200 publications per year, with the annual publication count exceeding 1000 in both 2022 and 2023. These results indicate an increasing level of interest and contributions from scholars in this specific field. Undoubtedly, the development and application of new technologies such as high-throughput sequencing and metagenomics play an important role in promoting the rise in this field [58,59]. Some countries or organizations have successively implemented projects related to gut microbiota and made breakthrough progress, which has also provided guidance and laid the foundation for further revealing the relationship between gut microbiota and intestinal diseases. For example, the National Institutes of Health of the USA released the Human Microbiome Project (HMP) in 2007, and the European Commission announced the launch of the Human Gut Metagenome Project in 2008.

China is the main contributor in the field of gut microbiota and intestinal diseases, followed by the USA, with a much higher number of publications than other countries. We found that 12 out of the top 20 institutions with the highest publication frequency belong to China, and 3 out of the top 5 institutions with the highest centrality belong to China. This further highlights China’s dominant position in the field of gut microbiota and intestinal diseases, indicating that China’s attention and importance to this field are increasing. It is worth noting that the Chinese Academy of Sciences is the institution with the largest number of publications, ranking fifth in terms of centrality, which means that the Chinese Academy of Sciences has the highest contribution and plays a leading role in this field.

From the perspective of journals published in the field of gut microbiota and intestinal diseases, researchers mainly focus on *Frontiers in Microbiology*, *Nutrients*, *Frontiers in Immunology*, *Frontiers in Cellular and Infection Microbiology*, *International Journal of Molecular Sciences*, *Gut Microbes*, *Scientific Reports*, *Plos One*, *Food & Function*, *Microorganisms*, and other journals. The top 20 journals belong to Q1 and Q2 of the Journal Citation Reports (16 in Q1) and 9 journals in the TOP journals, with an average impact factor of 6.4. From the perspective of cited journals, *Plos One*, *Nature*, *Gut*, *Proceedings of The National Academy of Sciences of The United States of America*, *Science*, *Scientific Reports*, *Gastroenterology*, *Cell*, *Frontiers in Microbiology*, and *Cell Host & Microbe* are the top 10 most cited journals. This series of results indicate that most publications in this field choose to be published in high-impact and high-quality research journals, indirectly reflecting the recognized importance and high research level of this field. It is worth noting that *Frontiers in Microbiology*, *Nutrients*, *Frontiers in Immunology*, *Gut Microbes*, *Scientific Reports*, *Plos One*, *Gastroenterology*, *World Journal of Gastroenterology*, and *Microbiome* are not only the top 20 journals with the most publications, but also the top 20 most cited journals, implying the importance of these 9 journals in the field of gut microbiota and intestinal diseases. In addition, Chinese scholars led by Zhang Xin have formed a prolific group of authors in this field. Turnbaugh PJ is the most frequently cited author.

Among the top 20 cited references, 6 are studies on the composition of gut microbiota in human health or diseases. The current epidemiological, pathological, omics, cellular, and animal research results reveal that gut microbiota to a considerable extent mediates human metabolic health and disease risk, and disrupted gut microbiota can lead to the occurrence of various intestinal diseases [60]. The seven cited references study how to regulate gut microbiota metabolites to affect host gut health. In recent years, the metabolites and functional mechanisms derived from gut microbiota have gradually been revealed. Relevant metabolites serve as signaling molecules and substrates for metabolic reactions in the body, affecting host physiology, pathology, and other processes. At present, there are numerous types of metabolites derived from gut microbiota, which can be roughly divided into three categories based on their sources and synthesis: (1) metabolites produced by gut microbiota from dietary components, such as short-chain fatty acids, tryptophan metabolites, trimethylamine oxides, and carbohydrates; (2) metabolites produced by the host and modified by gut microbiota, such as secondary bile acids; (3) metabolites that can be re-synthesized by gut microbiota, such as bacterial vitamins, polyamines, branched-chain amino acids, lipids, and neurotransmitters [61]. The two cited references study on the role of gut microbiota in the gut–brain axis. Gut microbiota is considered a key participant in the communication between the gut–brain axis. An increasing amount of evidence reveals bidirectional communication between the gut microbiome and the central nervous system, known as the “microbiota-gut–brain axis” [62]. Gut microbiota can regulate the immune system, vagus nerve, enteric nervous system, neuroendocrine system, and circulatory system by producing neuroactive substances, metabolites, and hormones. Furthermore, it participates in the complex regulation of social behavior, depressive like behavior, physical performance, and motivation, among other human health and diseases [63,64]. There is one cited reference on the application of Quantitative Insights Into Microbial Ecology 2 (QIIME 2) tool in gut microbiome data. QIIME is the most widely used analytical process in the field of microbiome and has been named one of the 25 milestone events in human microbiome research in the past 70 years by *Nature* in 2019. To meet the increasingly large scale of data and the demand for repeatable and traceable analysis, Professor Gregory Caporaso from Northern Arizona University led the development of a new QIIME 2 analysis platform from scratch, in collaboration with 112 peers from 79 institutions around the world [36]. It is a powerful, scalable, and decentralized new microbiome analysis platform that has been recognized as the analytical standard in the field of microbiome.

Keyword analysis can reflect the research hotspots and frontiers in the field of gut microbiota and intestinal diseases [65]. We conducted a systematic analysis of keyword co-occurrence network, clustering, and bursts. Gut microbiota, inflammation, ulcerative colitis, metabolism, obesity, fecal microbiota, diversity, mice, and Crohn’s disease are among the top 20 keywords with the highest frequency and centrality, representing the research hotspots in this field. Among the 15 keyword clusters, 9 clusters are related to the relationship between gut microbiota and host intestinal diseases, and have lasted for a long time, reflecting scholars’ continuous attention and research on the interaction mechanism between gut microbiota and intestinal diseases, such as dendritic cells, short chain fatty acids, intestinal barrier, gut–brain axis, and symbiotic bacteria. In addition, the keyword bursts analysis indicates that the field has gradually shifted its focus from the relationship between single intestinal diseases and the diversity of gut microbiota, to the role of specific and symbiotic gut microbiota in intestinal diseases, and then to the application of gut microbiota in the treatment and prevention of gut health such as high-fat diet. In other words, to gain a mechanistic understanding of the impact of gut microbiota on host gut diseases, researchers have shifted their focus from traditional descriptive analysis of gut microbiota composition to exploring causal relationships [56]. Therefore, we believe that there are the following research trends in the field of gut microbiota and intestinal diseases: (1) The correlation between specific intestinal diseases and gut microbiota diversity, such as irritable bowel syndrome, inflammatory bowel disease, celiac disease, and colorectal cancer. (2) The mechanism of involvement of gut microbiota in intestinal diseases, such as short-chain fatty acids, intestinal barrier, and gut–brain axis. (3) Exploration of important gut microbiota related to intestinal diseases, such as probiotics, harmful bacteria, and opportunistic pathogens. (4) The relationship between gut microbiota and human intestinal health, such as obesity, diabetes, hypertension, and cancer.

## 5. Strengths and Limitations

The massive and complex literature makes it difficult to quantify the research process and macroscopic patterns in specific fields. Bibliometrics is the means to solve this problem, which is a research method that uses quantitative analysis to examine the scientific literature, relying on mathematical, statistical, and bibliometric techniques. We have intuitively and systematically revealed the research status, hotspots, and trends in the field of gut microbiota and intestinal diseases through bibliometric visualization analysis. However, our research may still have some limitations. Firstly, the data only comes from a single source, namely the WoSCC database, resulting in the omission of publications from other databases. Secondly, the publication types are limited to articles and reviews, and do not include other types of studies such as conference reports and books. Finally, only English publications were included in this study, which may have overlooked other non-English publications such as Chinese and French. Nevertheless, we believe that this study adequately reflects the overall situation in the field of gut microbiota and intestinal diseases.

## 6. Conclusions

This is the first comprehensive bibliometric and statistical analysis of the field of gut microbiota and intestinal diseases. In this study, we found that the number of annual publications in this field has been increasing year by year and showing an upward trend, indicating that this field is receiving increasing attention globally. China is the country with the largest number of publications. Chinese institutions occupy the largest number of seats among the top 20 most productive institutions, and the Chinese Academy of Sciences is the institution with the largest number of publications. China has made significant contributions to the progress in this field. The cooperation between different countries and institutions is weak, and it is necessary to further strengthen close cooperation. *Frontiers in Microbiology*, *Nutrients*, and *Frontiers in Immunology* are the journals with the most publications, while *Plos One*, *Nature*, and *Gut* are the journals with the most citations. Chinese scholars led by Zhang Xin have formed a prolific group of authors in this field. Turbbaugh PJ is the author with the highest citation frequency. Among the top 20 most frequently cited references, the research mainly focuses on the composition of gut microbiota in human health or diseases, the interaction between gut microbiota and inflammatory bowel disease, the regulation of gut microbiota metabolites affecting host gut health, the role of gut microbiota in the gut–brain axis, and the application of QIIME 2 tool in gut microbiome data. Keyword analysis shows that the field has shifted from focusing on traditional descriptive analysis of gut microbiota composition to focusing on the role of specific and symbiotic gut microbiota in intestinal diseases, and then focusing on the application of gut microbiota in the treatment and prevention of gut health such as high-fat diets. In addition, the correlation between specific intestinal diseases and gut microbiota diversity, the mechanism of gut microbiota involvement in intestinal diseases, the exploration of important gut microbiota related to intestinal diseases, and the relationship between gut microbiota and human gut health will be future research hotspots and trends in the field of gut microbiota and intestinal diseases.

## Figures and Tables

**Table 1:** Top 20 countries/regions in the gut microbiota and intestinal diseases field.

Rank	Freq	Centrality	Countries/Regions
1	2818	0.15	China
2	1187	0.15	USA
3	350	0.21	Italy
4	241	0	Germany
5	208	0.04	Japan
6	202	0	Canada
7	200	0.5	France
8	199	0.23	United Kingdom
9	182	0.19	Spain
10	149	0	Netherlands
11	126	0.33	Australia
12	125	0	South Korea
13	113	0.04	Brazil
14	102	0.15	India
15	96	0	Poland
16	86	0.27	Sweden
17	86	0	Belgium
18	76	0.16	Switzerland
19	62	0.43	Denmark
20	61	0.03	Ireland

**Table 2:** Top 20 institutions in the gut microbiota and intestinal diseases field.

Rank	Freq	Centrality	Institution	Country
1	154	0.24	Chinese Academy of Sciences	China
2	147	0.23	University of California System	USA
3	111	0.25	Institut National de la Sante et de la Recherche Medicale (INSERM)	France
4	110	0.33	Ministry of Agriculture and Rural Affairs	China
5	102	0.01	Zhejiang University	China
6	95	0.07	Harvard University	USA
7	91	0.01	French National Research Institute for Agriculture, Food and Environment (INRAE)	France
8	81	0.05	Chinese Academy of Medical Sciences—Peking Union Medical College	China
9	78	0.01	Shanghai Jiao Tong University	China
10	76	0.1	Sun Yat Sen University	China
11	75	0.31	Université Paris Cité	France
12	73	0.24	Harvard Medical School	USA
13	71	0.08	Southern Medical University	China
14	71	0.13	China Agricultural University	China
15	69	0.18	University of Chinese Academy of Sciences	China
16	65	0	Jilin University	China
17	60	0.11	Centre National de la Recherche Scientifique (CNRS)	France
18	59	0.03	Catholic University of the Sacred Heart	Italy
19	58	0.3	Chinese Academy of Agricultural Sciences	China
20	57	0.08	Capital Medical University	China

**Table 3:** Top 20 journals in the gut microbiota and intestinal diseases field.

Rank	Journal	Number of Publications	Annual Number of All Publications	IF (2024)	Category Quartile	Top Journal	JCR Category	Country
1	*Frontiers in Microbiology*	198	3803	4.0	Q2	Yes	Microbiology	Switzerland
2	*Nutrients*	180	4930	4.8	Q1	No	Nutrition and Dietetics	Switzerland
3	*Frontiers in Immunology*	179	5971	5.7	Q1	No	Immunology	Switzerland
4	*Frontiers in Cellular and Infection Microbiology*	132	1343	4.6	Q1	No	Microbiology	Switzerland
5	*International Journal of Molecular Sciences*	121	17,106	4.9	Q1	No	Biochemistry and Molecular Biology	Switzerland
6	*Gut Microbes*	104	307	12.2	Q1	Yes	Microbiology	USA
7	*Scientific Reports*	103	22,037	3.8	Q1	No	Multidisciplinary Sciences	United Kingdom
8	*Plos One*	102	14,545	2.9	Q1	No	Multidisciplinary Sciences	USA
9	*Food & Function*	91	758	5.1	Q1	Yes	Biochemistry and Molecular Biology	United Kingdom
10	*Microorganisms*	87	2904	4.1	Q2	No	Microbiology	Switzerland
11	*Frontiers in Nutrition*	76	1764	4.0	Q2	No	Nutrition and Dietetics	Switzerland
12	*Frontiers in Pharmacology*	75	2944	4.4	Q1	No	Pharmacology and Pharmacy	Switzerland
13	*Journal of Agricultural and Food Chemistry*	64	1808	5.7	Q1	Yes	Agriculture, Multidisciplinary	USA
14	*Biomedicine & Pharmacotherapy*	55	1743	6.9	Q1	Yes	Medicine, Research and Experimental	France
15	*World Journal of Gastroenterology*	55	411	4.3	Q1	No	Gastroenterology and Hepatology	China
16	*Microbiome*	49	262	13.8	Q1	Yes	Microbiology	United Kingdom
17	*Microbiology Spectrum*	43	2164	3.7	Q2	No	Microbiology	USA
18	*Gastroenterology*	41	165	25.7	Q1	Yes	Gastroenterology and Hepatology	USA
19	*Aquaculture*	38	1059	3.9	Q1	Yes	Marine and Freshwater Biology	Netherlands
20	*Fish & Shellfish Immunology*	38	688	4.1	Q1	Yes	Marine and Freshwater Biology	United Kingdom

**Table 4:** Information on gut microbiota associated with specific intestinal diseases.

Intestinal Disease	Bacteria	Phylum	Study Method	References
Irritable bowel syndrome (IBS)	*Bacteroides dorei*	Bacteroidetes	16S rRNA sequencing, metatranscriptomics	[39]
	*Ruminococcus gnavus*	Firmicutes	16S rRNA sequencing	[40]
	Genera *Coprococcus*, *Collinsella*, and *Coprobacillus*	Firmicutes, Actinobacteriota	16S rRNA sequencing	[41]
	Genera *Dorea*, *Ruminococcus*, *Clostridium*, and Faecalibacterium	Firmicutes, Bacteroidetes, Actinobacteriota	Reproducible phylogenetic microarray	[42]
	Family Enterobacteriaceae, family Lactobacillaceae, genus *Bacteroides*, uncultured Clostridiales I, genus *Faecalibacterium* (including *Faecalibacterium prausnitzii*), and genus *Bifidobacterium*	Proteobacteria, Firmicutes, Bacteroidota, Actinobacteriota	Systematic review	[43]
Inflammatory bowel diseases (IBD)	*Faecalibacterium prausnitzii*	Firmicutes	In vitro (cellular models) and vivo colitis in mice	[44]
	*Escherichia coli*	Proteobacteria	16S rRNA sequencing	[45]
	*Genus Fusobacterium*	Fusobacteria	Systematic review	[46]
	Family Enterobacteriaceae	Firmicutes, Proteobacteria	16S rRNA sequencing	[47]
Celiac disease (CD)	*Dialister invisus*, genus *Parabacteroides*, Family Lachnospiraceae	Firmicutes, Bacteroidetes	Metagenomic sequencing	[48]
Colorectal cancer (CRC)	*Fusobacterium nucleatum*	Fusobacteria	16S rRNA sequencing	[49]
	*Morganella morganii*	Proteobacteria	Multi omics analysis	[50]
	*Fusobacterium nucleatum*, *Escherichia coli*, *Bacteroides fragilis*	Fusobacteria, Proteobacteria, Bacteroidetes	Systematic review	[51]
Antibiotic-associated diarrhea (AAD)	Genera *Enterococcus*, *Eubacterium*, *Ruminococcus*, and *Blautia*	Firmicutes	16S rRNA sequencing	[52]
	Genera *Akkermansia*, *Alistipes*, and *Actinomyces*	Verrucomicrobia, Bacteroidetes, and Actinobacteriota	16S rRNA sequencing	[53]

**Table 5:** Top 20 cited authors in the gut microbiota and intestinal diseases field.

Rank	Freq	Centrality	Cited Author
1	887	0.71	Turnbaugh PJ
2	799	0.54	Cani PD
3	771	0.92	Qin JJ
4	700	0.11	Ley RE
5	654	0.09	Backhed F
6	642	0.18	Caporaso JG
7	533	0.14	Edgar RC
8	460	0.05	Hooper LV
9	439	0.2	Segata N
10	436	0.88	Sokol H
11	431	0.49	Atarashi K
12	419	0.23	David LA
13	409	0.14	Everard A
14	370	0.3	Eckburg PB
15	370	0.48	Round JL
16	317	0.29	Frank DN
17	315	0.39	Furusawa Y
18	314	0.73	Arumugam M
19	307	0	Wang Q
20	306	0.03	Wang Y

**Table 6:** Top 20 cited journals in the gut microbiota and intestinal diseases field.

Rank	Freq	Centrality	Cited Journal	IF (2024)
1	3979	0.32	*Plos One*	2.9
2	3934	0.31	*Nature*	50.5
3	3413	0.1	*Gut*	23.0
4	3344	0.03	*Proceedings of The National Academy of Sciences of The United States of America*	9.4
5	3144	0.08	*Science*	44.7
6	3039	0.14	*Scientific Reports*	3.8
7	2953	0.18	*Gastroenterology*	25.7
8	2740	0.07	*Cell*	45.5
9	2431	0	*Frontiers in Microbiology*	4.0
10	2383	0	*Cell Host & Microbe*	20.6
11	2269	0	*Gut Microbes*	12.2
12	2241	0.01	*Nature Communications*	14.7
13	2136	0.13	*Nutrients*	4.8
14	2031	0.21	*Applied and Environmental Microbiology*	3.9
15	1977	0	*Nature Reviews Gastroenterology & Hepatology*	45.9
16	1929	0	*Frontiers in Immunology*	5.7
17	1927	0	*Nature Medicine*	58.7
18	1918	0	*World Journal of Gastroenterology*	4.3
19	1794	0	*Microbiome*	13.8
20	1609	0	*Lancet*	98.4

**Table 7:** Top 20 cited references in the gut microbiota and intestinal diseases field.

**Rank**	**Freq**	**Centrality**	**Author**	**Year**	**Title**	**Journal**	**DOI**
1	165	0	Venegas DP	2019	Short Chain Fatty Acids (SCFAs)-Mediated Gut Epithelial and Immune Regulation and Its Relevance for Inflammatory Bowel Diseases	*Frontiers in Immunology*	10.3389/fimmu.2019.00277
2	158	0.07	Bolyen E	2019	Reproducible, interactive, scalable and extensible microbiome data science using QIIME 2	*Nature Biotechnology*	10.1038/s41587-019-0209-9
3	156	1.25	David LA	2014	Diet rapidly and reproducibly alters the human gut microbiome	*Nature*	10.1038/nature12820
4	136	0.22	Rinninella E	2019	What is the Healthy Gut Microbiota Composition? A Changing Ecosystem across Age, Environment, Diet, and Diseases	*Microorganisms*	10.3390/microorganisms7010014
5	123	0.02	Koh A	2016	From Dietary Fiber to Host Physiology: Short-Chain Fatty Acids as Key Bacterial Metabolites	*Cell*	10.1016/j.cell.2016.05.041
6	119	0.25	Lavelle A	2020	Gut microbiota-derived metabolites as key actors in inflammatory bowel disease	*Nature Reviews Gastroenterology & Hepatology*	10.1038/s41575-019-0258-z
7	117	0.11	Lloyd-Price J	2019	Multi-omics of the gut microbial ecosystem in inflammatory bowel diseases	*Nature*	10.1038/s41586-019-1237-9
8	109	0.03	Fan Y	2021	Gut microbiota in human metabolic health and disease	*Nature Reviews Microbiology*	10.1038/s41579-020-0433-9
9	108	0.5	Thursby E	2017	Introduction to the human gut microbiota	*Biochemical Journal*	10.1042/BCJ20160510
10	106	0.13	Furusawa Y	2013	Commensal microbe-derived butyrate induces the differentiation of colonic regulatory T cells	*Nature*	10.1038/nature12721
11	105	0.24	Yatsunenko T	2012	Human gut microbiome viewed across age and geography	*Nature*	10.1038/nature11053
12	104	0.21	Dalile B	2019	The role of short-chain fatty acids in microbiota-gut–brain communication	*Nature Reviews Gastroenterology & Hepatology*	10.1038/s41575-019-0157-3
13	103	0.08	Smith PM	2013	The microbial metabolites, short-chain fatty acids, regulate colonic Treg cell homeostasis	*Science*	10.1126/science.1241165
14	102	0.07	Gevers D	2014	The treatment-naive microbiome in new-onset Crohn’s disease	*Cell Host & Microbe*	10.1016/j.chom.2014.02.005
15	102	0.07	Ni J	2017	Gut microbiota and IBD: causation or correlation?	*Nature Reviews Gastroenterology & Hepatology*	10.1038/nrgastro.2017.88
16	101	0.11	Cryan JF	2019	The Microbiota-Gut–Brain Axis	*Physiological Reviews*	10.1152/physrev.00018.2018
17	98	0.39	Agus A	2018	Gut Microbiota Regulation of Tryptophan Metabolism in Health and Disease	*Cell Host & Microbe*	10.1016/j.chom.2018.05.003
18	94	0.47	Qin JJ	2012	A metagenome-wide association study of gut microbiota in type 2 diabetes	*Nature*	10.1038/nature11450
19	94	0.09	Franzosa EA	2019	Gut microbiome structure and metabolic activity in inflammatory bowel disease	*Nature Microbiology*	10.1038/s41564-018-0306-4
20	93	0.02	Lynch SV	2016	The Human Intestinal Microbiome in Health and Disease	*New England Journal of Medicine*	10.1056/NEJMra1600266

## Data Availability

The original contributions presented in this study are included in the article. Further inquiries can be directed to the corresponding author.
